# Evidence of necroptosis in osteoarthritic disease: investigation of blunt mechanical impact as possible trigger in regulated necrosis

**DOI:** 10.1038/s41419-019-1930-5

**Published:** 2019-09-17

**Authors:** Jana Riegger, Rolf E. Brenner

**Affiliations:** Division for Biochemistry of Joint and Connective Tissue Diseases, Department of Orthopedics, University of Ulm, Ulm, Germany

**Keywords:** Necroptosis, Target identification, Trauma

## Abstract

Joint injuries are highly associated with cell death and development of posttraumatic osteoarthritis (PTOA). The present study focused on necroptosis as a possible modality of chondrocyte death after cartilage trauma and its relevance in OA disease in general. For this purpose, apoptosis- and necroptosis-associated markers were determined in highly degenerated (ICRS ≥ 3) as well as macroscopically intact cartilage tissue (ICRS ≤ 1) by means of real-time PCR and immunohistochemistry (IHC). Moreover, influence of blunt trauma and/or stimulation with cycloheximide (CHX), TNF-a, and caspase-inhibitor zVAD were investigated in cartilage explants (ICRS ≤ 1). Further characterization of necroptosis was performed in isolated chondrocytes. We found that gene expression levels of RIPK3 (4.2-fold, *P* < 0.0001) and MLKL (2.7-fold, *P* < 0.0001) were elevated in highly degenerated cartilage tissue, which was confirmed by IHC staining. After ex vivo trauma and/or CHX/TNF stimulation, addition of zVAD further enhanced expression of necroptosis-related markers as well as release of PGE2 and nitric oxide, which was in line with increased cell death and subsequent release of intracellular HMGB1 and dsDNA in CHX/TNF stimulated chondrocytes. However, trauma and/or chemically induced cell death and subsequent release of pro-inflammatory mediators could be largely attenuated by RIPK1-inhibitor necrostatin 1 or antioxidant N-acetylcysteine. Overall, the study provided clear evidence of necroptotic cell death in OA disease. Moreover, a possible link between cartilage injury and necroptotic processes was found, depending on oxidative stress and cytokine release. These results contribute to further understanding of cell death in PTOA and development of novel therapeutic approaches.

## Introduction

Cartilage trauma is known to result in induction of catabolic gene expression, inflammation, and progressive cell death. In the long term, these trauma-induced pathomechanisms drive the proceeding degradation of the extracellular matrix (ECM) and are thus considered as the main risk factors for the development of posttraumatic osteoarthritis (PTOA). By means of a human ex vivo cartilage trauma model, we have previously demonstrated that we are able to reproduce early pathogenetic processes after a single-impact cartilage injury, allowing investigation of the pathogenesis as well as testing of possible treatment strategies^1–3^.

However, to attenuate the ongoing cell loss after cartilage trauma more profound pathophysiologic knowledge with respect to the underlying mechanisms and, in the first place, the identification of the correct modality of cell death, is required. On one hand, the mechanical impact and the subsequent compression of the ECM leads to an immediate necrosis of the embedded cells. On the other hand, there are complex molecular pathways, primarily provoked by extrinsic stimuli, deciding upon survival or subsequent regulated cell death^4^. Typical death-associated triggers are pro-inflammatory tumor necrosis factor alpha (TNF-a), reactive oxygen/nitrogen species (ROS/RNS) or damage associated molecular patterns (DAMPs), which can be all associated with traumatic tissue injury^5,6^.

Besides apoptosis, a second form of regulated cell death, the so-called necroptosis, has been described more recently and has caught a lot of attention since then^7^. In fact, the modality of cell death plays a more important role as it might seem at first glance. In general, apoptosis can be considered as the “clean” way to eliminate malfunctioning cells, which does not entail release of intracellular contents and thus prevents immunological response. On the contrary, necroptosis is the “dirty” execution, leading to bursting of the cell membrane and causing detrimental effects in the surrounding tissues^8^. According to the current state of scientific knowledge, it is assumed that necroptotic cell death promotes inflammation and progression of degenerative disease^9^.

On molecular level, necroptosis and apoptosis have a common origin: the activation of the TNF receptor 1 (TNFR1) and the formation of the TNFR1 complex I, including the receptor interacting protein kinase 1 (RIPK1). The decisive aspect that determines the mode of regulated cell death is the presence or absence of caspase 8 (CASP8) activity. This key enzyme executes apoptosis by inducing the caspase cascade^10^. Deactivation of CASP8, i.e., by pan caspase-inhibitor zVAD-FMK, has been shown to pave the way for the necroptotic pathway, which is initiated by interaction of RIPK1 and RIPK3 resulting in the so-called necrosome (complex IIB)^11^. Subsequent recruitment and phosphorylation of mixed lineage kinase like (MLKL), finally leading to disruption of the membrane integrity, possibly by pore formation and subsequent calcium influx^12^. Degterev et al. identified Necrostatin 1 (Nec-1) as a reliable small-molecule inhibitor of necroptotic cell death^7,13^. Moreover, Nec-1 has additionally been found to prevent RIP1-dependent apoptosis and autophagy after traumatic injury^14,15^.

According to Zhang et al., necroptosis might also play a role in mandibular cartilage thinning in the course of repetitive mechanical loading over 4–7 days and was thought to be driven by ROS generation as demonstrated in a rat model^16^. However, many questions about trauma-induced necroptosis and its relevance in PTOA development remain unclear. Therefore, we investigated the presence as well as possible consequences of necroptosis after single-impact cartilage trauma and tested different therapeutic approaches to prevent chondrocytes from cell death.

## Material and methods

### Preparation and cultivation of human cartilage explants

Human cartilage was obtained from donors undergoing total knee joint replacement due to osteoarthritic (OA) disease. Informed consent was obtained from all patients according to the Declaration of Helsinki and the guidelines of the Ethical Committee of the University of Ulm. Only macroscopically intact tissue samples (International Cartilage Repair Society (ICRS) grade ≤ 1)^17^ from femoral condyles were used in the ex vivo cartilage trauma model as well as for chondrocyte isolation. Full-thickness cartilage explants (*Ø* = 6 mm) were cultivated in serum-containing medium (1:1 DMEM/Ham’s F12, 10% fetal bovine serum, 0.5% penicillin/streptomycin (PAA Laboratories, Pasching, Austria), 0.5% l-glutamine and 10 μg/ml 2-phospho-L-ascorbic acid trisodium salt (Sigma-Aldrich, Taufkirchen, Germany)) at 37 °C, 5% CO_2_, and 95% humidity until traumatization on the following day. During the running experiment, the explants were cultivated in serum-free medium (DMEM, 1% sodium pyruvate, 0.5% l-glutamine, 1% nonessential amino acids, 0.5% penicillin/streptomycin, and 0.1% insulin-transferrin-sodium selenite (ITS, Sigma-Aldrich). All medium components were purchased from Biochrom (Berlin, Germany) unless specified otherwise.

### Collection of highly degenerated human cartilage tissue

Highly degenerated human cartilage tissue (ICRS grade ≥ 3) was additionally collected from the same source as mentioned above. Tissue sections of 13 patients (mean age 65 years, ranging 54–75 years) were washed with PBS and immediately stored in liquid nitrogen for RNA isolation or formalin for IHC, without in vitro culturing.

### Impact loading and subsequent treatment

Cartilage explants from tissue of 27 patients (mean age 68 years, ranging 54–78 years) were subjected to a defined impact energy of 0.59 J by using a drop-tower model as previously described^1–3^. In addition, we included a chemical induction by TNF-a (10 or 100 ng/mL; Gibco, Thermo Fisher Scientific, Schwerte, Germany) and simultaneous sensitization with translation inhibitor cycloheximide (CHX; 5 or 10 µg/mL; Sigma-Aldrich), as described in previous studies^18,19^. To distinguish between necroptotic and apoptotic processes, cartilage explants were continuously treated with 2 mM N-acetyl-L-cysteine (Sigma-Aldrich)^1^ or 40 µM Nec-1 (Sigma-Aldrich) w/ and w/o and 20 µM zVAD-FMK (zVAD; R&D Systems, Wiesbaden, Germany) for 4 days. MLKL inhibitor Necrosulfonamide (NSA; Tocris Bioscience, Bristol, UK) was exemplarily tested at a concentration of 2.5 µM. Fresh additives were provided after 24 h. Optimum concentration of Nec-1 was evaluated in prior cell culture experiments (Supplementary Fig. S1).

### Live/Dead Cell Cytotoxicity Assay

The percentage of viable cells was evaluated by using the Live/Dead Viability/Cytotoxicity Kit (Thermo Fisher Scientific). Unfixed tissue sections (0.5-mm thickness) were stained with 1 mM calcein acetoxymethyl ester (calcein-AM) and 2 mM ethidium homodimer-1 (EthD-1) for 30 min. After washing in PBS, stained tissue sections were microscopically analysed by means of a Z-stack module (AxioVision software; Carl Zeiss) as previously described^1^.

### Immunohistochemistry (IHC)

For IHC, paraffin-embedded sections (3.5 μm) were dewaxed and rehydrated. Antigen retrieval was performed by incubation in sodium citrate buffer pH 6.0 at 65 °C overnight. After cooling, unspecific antigens were blocked with the DAKO blocking reagent (Dako, Glostrup, Denmark), followed by overnight incubation with the 1:250 diluted rabbit monoclonal antibody [EPR9514] against human p-MLKL (phospho S358; abcam, Cambridge, UK), human cleaved caspase 8 (NB100-56116; NOVUS Biologicals, Centennial, CO, USA) or RIPK3 (GTX107574; GeneTex, Irvine, CA, USA) at 4 °C. Sections were treated with 3% hydrogen peroxide before starting the staining with the Dako LSAB2 System-HRP kit (Dako, Glostrup, Denmark). In all samples a final staining of cell nuclei by Gill’s hematoxylin No 3 (Sigma-Aldrich) was performed. In case of cleaved CASP8 and p-MLKL, the percentage of positive cells was quantified by manual counting of the stained sections.

### Isolation and Cultivation of human Chondrocytes

Chondrocytes were enzymatically isolated from human cartilage of eight patients (mean age 68, range 54–83 years). In short, full-thickness cartilage was minced and digested for 45 min with 0.2% pronase (Sigma-Aldrich), followed by washing and a second digest with 0.025% collagenase (Sigma-Aldrich) overnight. After washing with PBS and filtration through a 40 µm cell strainer, cells (passage 0) were cultured in serum-containing medium (see above). Chondrocytes were split at a confluence of 80% and used in passage 1 or 2.

### Stimulation and microscopic analysis of isolated Chondrocytes

Chondrocytes were seeded on 48-well cell culture plates for assessment of the cytotoxicity and 24-well cell culture plates for RNA extraction (0.25 × 10^5^ cells/cm^2^), respectively, in serum-containing chondrocyte medium. The following day, serum-containing medium was replaced by serum-reduced medium (1:1 serum-containing medium/serum-free medium; 5% FCS (v/v)) and cells were stimulated with trauma-conditioned medium (TCM), obtained from impacted cartilage explants (harvested 24 h after trauma), TNF/CHX with and without zVAD as described above. Morphologic changes in chondrocytes were documented after 24 h, by using phase-contrast microscopy (Zeiss, Oberkochen, Germany). Cells were lysed after 48 h for RNA isolation.

### Gene expression analysis

For total RNA extraction from cartilage tissue, cryopreserved explants were pulverized with a microdismembrator S (B. Braun Biotech, Melsungen, Germany), following RNA isolation by using the RNeasy Lipid Tissue Mini Kit. In case of monolayer cultured chondrocytes, RNA was isolated with the RNeasy Mini Kit (Qiagen, Hilden, Germany). Afterwards, reverse transcription was performed with the Omniscript RT Kit (Qiagen).

Relative gene expression levels of the target genes were determined by means of quantitative real-time polymerase chain reaction (qRT-PCR), using the 2^−ΔΔCt^ method of the StepOnePlus™ real-time PCR system (Applied Biosystems, Darmstadt, Germany). Target sequences were detected by using TaqMan^®^ Gene Expression Master Mix and the following TaqMan^®^ Gene Expression Assays (both Applied Biosystems): CASP3 Hs002343487_m1; CASP8 Hs01018151_m1, HPRT1 Hs02800695_m1, MLKL Hs04188505_m1, RIPK1 Hs01041869_m1, and RIPK3 Hs00179132_m1. In case of 18S rRNA and GAPDH, respectively, Power SYBR^®^ Green PCR Master Mix (Applied Biosystems) was used for 18S rRNA, 5′-CGCAGCTAGGAATAATGGAATAGG-3′ (forward) and 5′-CATGGCCTCAGTTCCGAAA-3′ (reverse), and Platinum^®^ SYBR^®^ Green qPCR SuperMix-UDG (Invitrogen) for GAPDH, 5′-TGGTATCGTGGAAGGACTCATG-3′ (forward) and 5′-TCTTCTGGGTGGCAGTGATG-3′ (reverse). Target mRNA-expression was normalized to the endogenous controls 18S rRNA, GAPDH, and HPRT1.

### AlamarBlue cell proliferation and cytotoxicity assay

Quantitative measurement of cell viability was attained by means of an alamarBlue assay (BioRad, Munich, Germany). The conversion of nonfluorescent resazurin to fluorescent resorufin can be considered as proportional to the number of living cells. After 24 h or 48 h stimulation culture medium was harvested and cells were incubated for 4 h with 200 µL of a 5% alamarBlue solution (in serum-reduced medium) at 37 °C. After the incubation period, the fluorescence intensities were detected at 550 nm excitation and 590 nm emission by using the multimode microplate reader Infinite M200 Pro (Tecan Austria GmbH Groedig, Austria). Blank values (5% alamarBlue solution in empty well) were subtracted from measured values, which were then normalized to the unstimulated control.

### Analysis of culture media

In case of the cartilage model, NO level was determined by quantification of nitrite, a stable end product of the NO metabolism, using a Griess assay (Griess Reagent System; Promega). The release of PGE2 was evaluated by means of an enzyme-linked immunosorbent assay (ELISA, Enzo Life Sciences Inc., Lause, Switzerland). The total amount of NO and PGE2, respectively, was relativized on the weight multiplied by cell viability of the corresponding cartilage explant as described before.^1^

For cell culture experiments, release of dsDNA and HMGB1, respectively, was detected by using Hoechst 33258 (Sigma-Aldrich) and a specific ELISA (Arigo biolaboratories Corp., Hsinchu City, Taiwan). HMGB1 release was normalized to the alamarBlue fluorescence intensity.

### Statistical analysis

Statistical analysis of results from at least three independent experiments (biological replicates) was performed by using GraphPad Prism version 6.0 h. Data sets with *n* ≥ 5 were tested for outliers with the Grubbs outlier test. Outliers were not included in statistical analyses. The applied statistic method can be found in the caption of the corresponding figures. Significant level was set to *α* = 0.05.

## Results

### Relevance of necroptosis in human OA disease

**Fig. 1:**
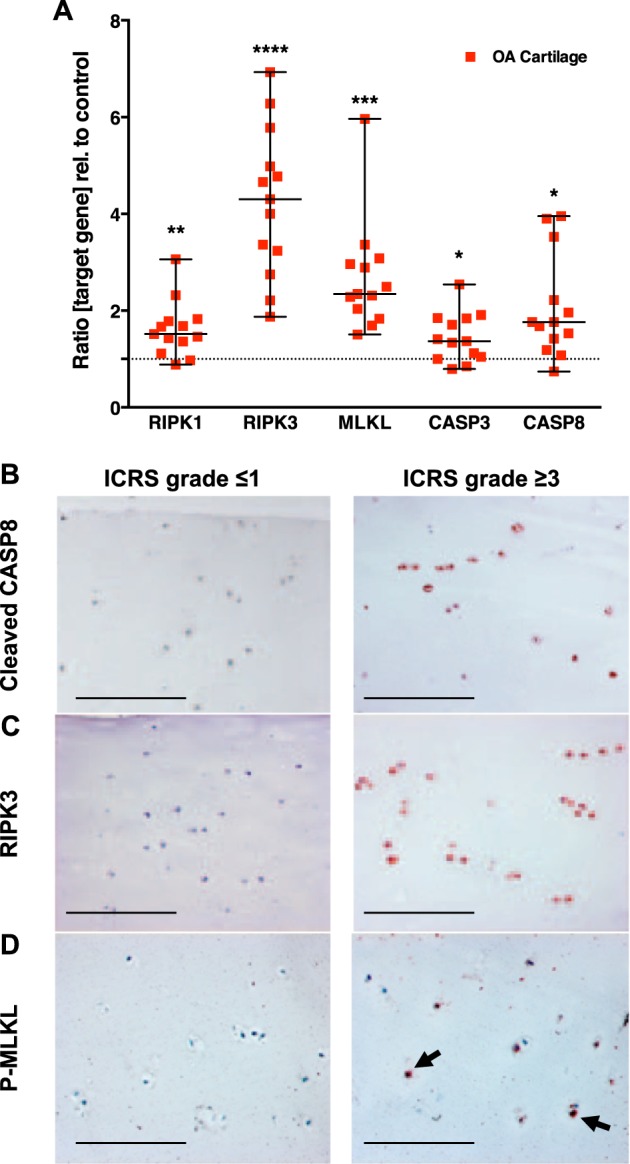
Expression of necroptosis- and apoptosis-related markers is elevated in OA cartilage. Necroptosis- and apoptosis-related markers in highly degenerated cartilage tissue of OA patients (ICRS grade ≥ 3) were determined by **a** gene expression analysis of apoptosis and necroptosis markers as well as immunohistochemical analysis (IHC) of **b** cleaved CASP8, **c** RIPK3, and **d** p-MLKL. Macroscopically intact cartilage (ICRS grade ≤ 1) served as control. Bars in the IHC images represent 200 µm. Statistical analysis was performed by an unpaired multiple *t* test; error bars indicate median and range from min to max; *n* = 13. Significant differences between groups were depicted as: *P < 0.05, **P < 0.01, ***P < 0.001, ****P < 0.0001

To estimate the relevance of necroptotic processes during the pathogenesis of OA disease, the gene expression of necroptosis- and apoptosis-associated markers was evaluated in highly degenerated tissue samples (ICRS grade ≥ 3). In fact, gene expression levels of necroptosis-associated RIPK1 (1.6-fold, *P* = 0.002), RIPK3 (4.2-fold, *P* < 0.0001), and MLKL (2.7-fold, *P* < 0.0001) as well as apoptosis-associated CASP3 (1.4-fold, *P* = 0.017) and CASP8 (1.8-fold, *P* = 0.012) were significantly higher as compared with that in macroscopically intact areas (ICRS grade ≤ 1) (Fig. 1a).

In accordance to the results of the gene expression analysis, cells of degenerated tissue samples were highly positive for RIPK3 and cleaved CASP8 (Fig. 1b, c). Moreover, p-MLKL, which can be considered as clear indication of necroptotic cell death, was notable evident in cells of highly degenerated cartilage (Fig. 1d). Overall, cleaved CASP8 was predominantly found in cells of the superficial zone, while p-MLKL-positive cells were rather located in the deep zone of the cartilage. Cells of macroscopically intact control tissue were slightly positive for cleaved CASP8, and did not exhibit RIPK3 and p-MLKL (Fig. 1b–f)

### Contribution of necroptosis in cell death after ex vivo cartilage trauma

Besides single mechanical impact (0.59 J), the effects of chemically induced necroptosis was investigated by using varying concentrations of TNF (10 or 100 ng/mL) and CHX (5 or 10 µg/mL) as well as different exposure times (“deprived” = exposure for the first 24 h; “continuously” = exposure during entire experiment (4 days)).

**Fig. 2:**
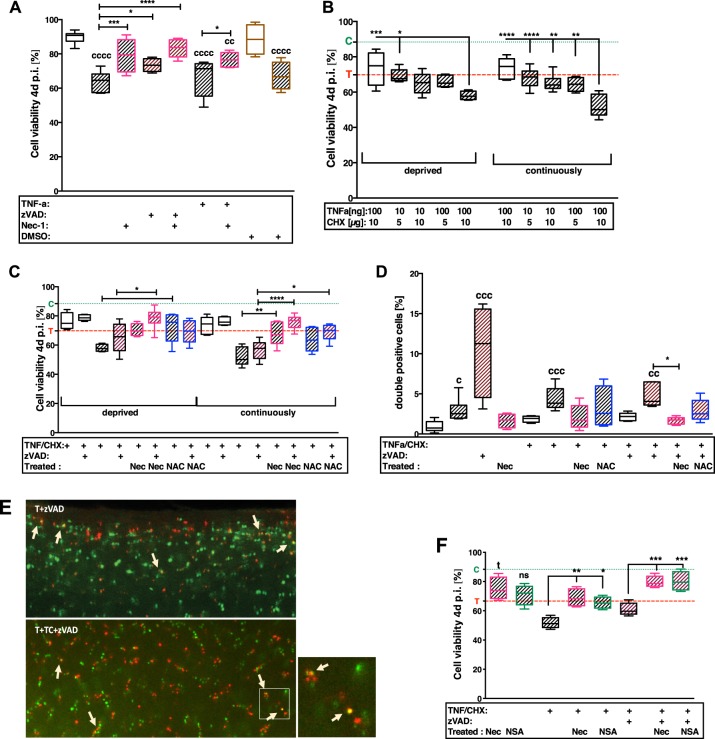
Treatment with NAC, Nec-1, and NSA, respectively, prevent from necroptotic cell death. After 4 days, cell viability was evaluated by Live/Dead staining in the following experimental approaches: **a** effects of trauma in absence/presence of TNF and treatment with zVAD or Nec-1; **b** titration of appropriate duration and concentration for chemical induction of necroptosis by TNF/CHX stimulation in impacted and unimpacted cartilage explants, respectively; **c** evaluation of therapeutic effects of Nec-1 or NAC after TNF/CHX stimulation with/ without co-stimulation by zVAD; **f** exemplary comparison of necroptosis inhibitors NSA and Nec-1, respectively. Statistical analysis was performed by **a, f** 1-way and **b, c** 2-way ANOVA, respectively, including a Bonferroni posttest (**a**–**c**: *n* ≥ 5; F: *n* ≥ 4). **d** Counting of double positive cells and statistical analysis, performed by Kruskal–Wallis test, including a Dunn’s posttest (*n* ≥ 4). **e** Exemplary fluorescence images of the live/dead analysis. Living cells exhibit a green fluorescence, dead cells a red one. Double positive cells appear in orange/yellow, due to overlay of both colors (exemplarily indicated by white arrows). Significant differences between groups were depicted as: [vs C] ^c^*P* < 0.05, ^cc^*P* < 0.01, ^ccc^*P* < 0.001, ^cccc^*P* < 0.0001; [vs T] ^t^*P* < 0.05; [between delineated groups] *P < 0.05, **P < 0.01, ***P < 0.001, ****P < 0.0001. Values are given as boxplots with median and whiskers (min to max); striped bars = impacted, blank bars = unimpacted; C (dotted line, green) = control level, T (dashed line, red) = trauma level

Traumatization of the cartilage explants resulted in significantly reduced cell viability ([vs C] −26%, *P* < 0.0001) (Fig. 2a). Treatment with Nec-1, zVAD and its combination, significantly increased the cell viability about 15.5% (*P* = 0.0005), 9.8% (*P* = 0.0393) and 19.7% (*P* < 0.0001), respectively.

Additional stimulation with TNF/CHX significantly enhanced the trauma effects depending on the concentration of the chemicals and the exposure time (Fig. 2b). Greatest effects were found after continuous stimulation with 100 ng/mL TNF and 10 µg/mL CHX. However, treatment with NAC or Nec-1 significantly protected the cartilage tissue from cell death after trauma plus TNF/CHX (Fig. 2c). Comparative evaluation between NAC and Nec-1 revealed that NAC was more effective after deprivation of TNF/CHX stimulation, while Nec-1 had higher cell-protective effects when the factors were continuously applied. The presence of zVAD clearly attenuated the cytotoxic effect of TNF/CHX and attained additive effects in combination with Nec-1 ([vs T + TNF/CHX] deprived: +13%, *P* = 0.019; continuously: +19.1%, *P* < 0.0001).

Moreover, frequent incidence of double stained cells was found after TNF/CHX stimulation with and without zVAD, as well as zVAD treatment of traumatized cartilage explants (Fig. 2d, e). This effect was considerably reduced after addition of Nec-1 and NAC, respectively. Treatment with MLKL inhibitor NSA (2.5 µM) exhibited comparable effects as found for Nec-1 (Fig. 2f). Although, cell-protective effects of NSA were not significant after trauma alone ([T vs. T + NSA]: +7.3%, *P* = 0.3), cell viability was significantly enhanced by NSA in combination with TNF/CHX with and without zVAD, implying a high incidence of necroptotic cell death due to TNF/CHX stimulation.

### Gene expression of necroptosis- and apoptosis-associated markers after ex vivo cartilage trauma

As presented in detail above, cartilage explants were exposed to different concentrations of TNF and CHX for 24 h (deprived) and 4 days (continuously), respectively. The results revealed that a continuous exposition to 100 ng/mL TNF and 10 µg/mL CHX was most suitable to achieve appropriate induction of necroptotic processes in our ex vivo trauma model (Supplementary Fig. S2). Therefore, the combined approaches with zVAD were performed under the above-mentioned conditions.

**Fig. 3:**
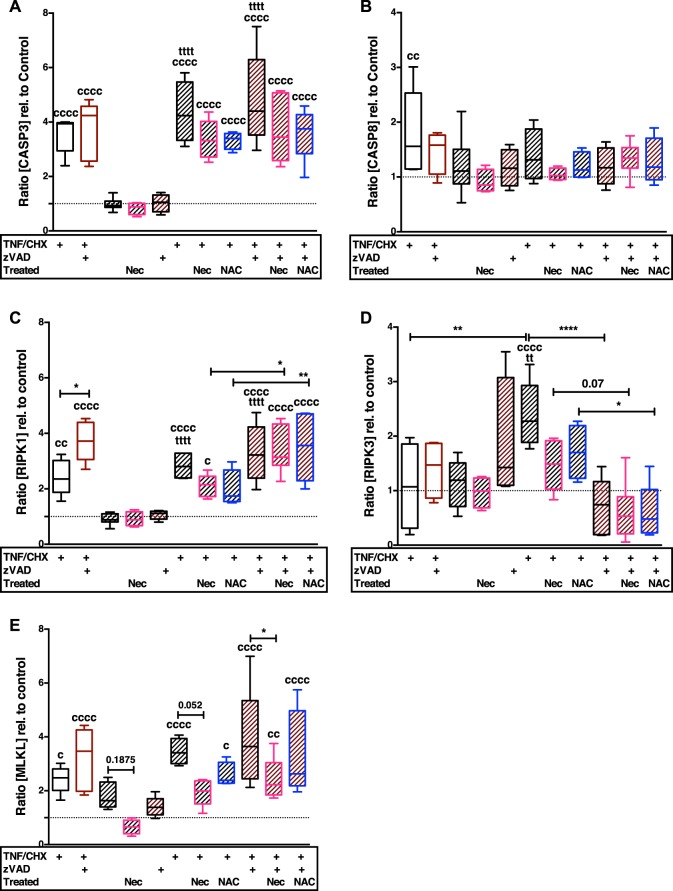
Enhanced gene expression of necroptosis- and apoptosis-related markers after TNF/CHX stimulation w/ and w/o zVAD can be attenuated by Nec-1 and NAC, respectively, to some extent. Gene expression analysis of **a** caspase 3, **b** caspase 8, **c** RIPK1, **d** RIPK3, and **e** MLKL necroptosis- and apoptosis-associated markers after trauma and/or stimulation with TNF/CHX w/ and w/o zVAD. Striped bars = impacted, blank bars = unimpacted. Statistical analysis was performed by one-way ANOVA, including a Bonferroni posttest (*n* ≥ 4). Significant differences between groups were depicted as: [vs C] ^c^*P* < 0.05, ^cc^*P* < 0.01, ^cccc^*P* < 0.0001; [vs T] ^tt^*P* < 0.01, ^tttt^*P* < 0.0001; [between delineated groups] *P < 0.05, **P < 0.01, ****P < 0.0001. Values are given as boxplots with median and whiskers (min to max); striped bars = impacted, blank bars = unimpacted

Mechanical impact alone only increased the gene expression of MLKL ([vs C] 1.8-fold, *P* = 0.185) (Fig. 3c). Addition of TNF/CHX with and without zVAD significantly induced the gene expression of CASP3 ([vs C] T + TC: 4.4-fold; T + TCZ: 4.8-fold, both *P* < 0.0001), RIPK1 ([vs C] T + TC: 2.8-fold; T + TCZ: 3.3-fold, both *P* < 0.0001), RIPK3 ([vs C] T + TC: 2-fold, *P* < 0.0001) and MKLK ([vs C] T + TC: 3.5-fold; T + TCZ: 4-fold, both *P* < 0.0001) (Fig. 3a, c–e). Surprisingly, addition of zVAD suppressed the TNF/CHX-induced gene expression of RIPK3 ([vs T + TC] −1.7-fold, *P* < 0.0001), while exhibiting rather inductive effect after trauma alone ([vs T] +0.8-fold, *P* = 0.1225). The gene expression of CASP8 was not significantly influenced after trauma with or without the additional stimuli (Fig. 3b).

The treatment with NAC or Nec-1 had largely suppressive effect on the gene expression of necroptosis and apoptosis markers.

### Correlation of pro-inflammatory markers and necroptosis after ex vivo cartilage trauma

**Fig. 4:**
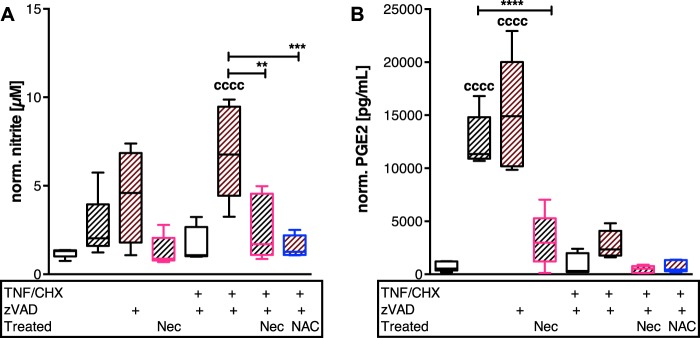
Release of inflammatory mediators is differently influenced by chemically induced necroptosis but can be attenuated by Nec-1 and NAC, respectively. Necroptosis was chemically induced in impacted and unimpacted cartilage explants by means of TNF/CHX w/ and w/o zVAD for 4 days (*n* ≥ 4). In addition, therapeutic effects of Nec-1 and NAC were evaluated in this context. Amounts of inflammatory markers **a** nitrite and **b** PGE2, respectively, were determined by Griess assay and ELISA, respectively. Statistical analysis was performed by one-way ANOVA, including a Bonferroni posttest. Significant differences between groups were depicted as: [vs C] ^cccc^*P* < 0.0001; [between delineated groups] **P < 0.01, ***P < 0.001, ****P < 0.0001. Values are given as boxplots with median and whiskers (min to max); striped bars = impacted, blank bars = unimpacted

As necroptosis is thought to provoke inflammatory processes in injured tissues, the nitrite level as well as PGE2 production was quantified (Fig. 4a, b). In fact, stimulation with TNF/CHX/zVAD significantly increased the release of NO into the culture medium ([vs C] 5.8-fold, *P* < 0.0001). This effect was suppressed by treatment with Nec-1 and NAC, respectively (−2.7-fold, *P* = 0.0021; and −4.4-fold, *P* = 0.0002, respectively). Although, PGE2 release was significantly enhanced after TNF/CHX stimulation as compared with the control (6.8-fold, *P* = 0.036), the amounts were strikingly lower as after trauma alone (19.4-fold, *P* < 0.0001). Moreover, zVAD treatment of traumatized cartilage explants resulted in elevated NO (1.7-fold) and PGE2 (1.2-fold) release, respectively. Treatment with Nec-1 or NAC significantly reduced the production of the tested metabolites.

### Presence of necroptosis- and apoptosis-associated markers in cartilage after ex vivo trauma

**Fig. 5:**
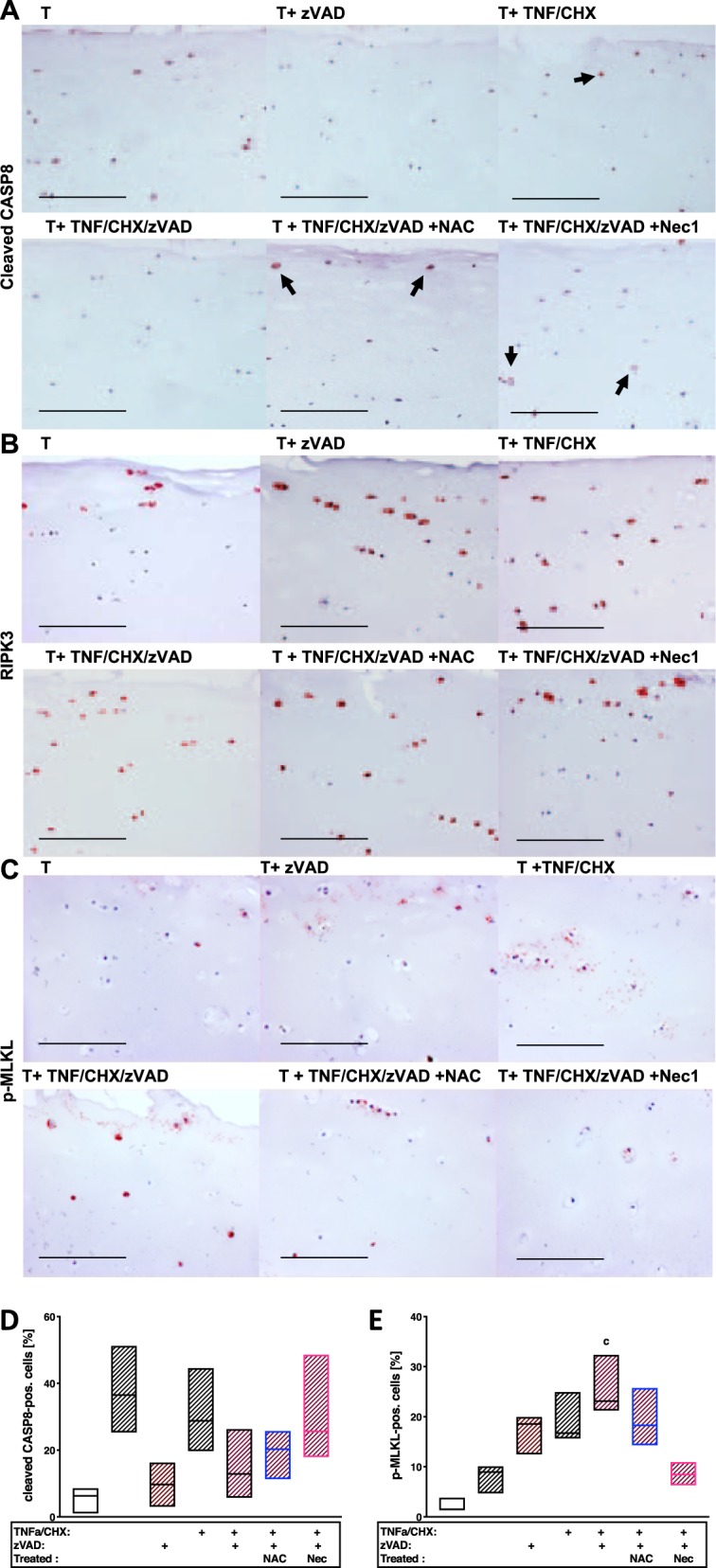
Necroptosis marker p-MLKL can be detected in OA cartilage and after TNF/CHX stimulation. Exemplary images of impacted and differently stimulated cartilage explants after immunohistochemical staining of **a** cleaved CASP8, **b** RIPK3, and **c** p-MLKL. In addition, percentage of cleaved CASP8− **(d)** and p-MLKL− **(e)** positive cells was quantified (*n* = 3). Images were acquired using a 20× objective; the bars represent 200 µm

Immunohistochemical staining revealed that the cells expressed both necroptosis as well as apoptosis markers after cartilage trauma (Fig. 5a–c). However, the percentage of cleaved CASP8-positive cells was clearly higher as compared with p-MLKL-positive ones (Fig. 5d, e). Moreover, a dissimilar distribution between cleaved CASP8 at the surface and p-MLKL in the lower part, which was also observed in highly degenerated cartilage, could be confirmed.

While co-stimulation with TNF/CHX significantly enhanced both necroptotic and apoptotic markers, addition of zVAD considerably reduced the presence of cleaved CASP8 but further enhanced the number of p-MLKL-positive cells. Addition of Nec-1, in contrast, diminished the percentage of p-MLKL-positive cells and seemed to promote CASP8 cleavage. NAC exhibited lower but still noticeable attenuating effects on MLKL phosphorylation after TNF/CHX/zVAD stimulation. In contrast to OA- or trauma-associated incidence of p-MLKL, chemically induced necroptosis was more pronounced in the upper parts of the cartilage.

### Characterization of necroptotic processes in isolated chondrocytes

Further in vitro investigation of necroptotic processes was performed on isolated chondrocytes in cell culture.

In accordance with the results of the ex vivo cartilage trauma model, gene expression of necroptosis- and apoptosis-associated proteins were significantly enhanced after TNF/CHX stimulation: RIPK1 ([vs C] 2.4-fold *P* = 0.0143), RIPK3 ([vs C] 2.9-fold *P* < 0.0001), MLKL ([vs C] 2.4-fold *P* = 0.0138), and CASP3 ([vs C] 2-fold *P* = 0.006). Nec-1 treatment suppressed the gene expression of RIPK1, RIPK3, and MLKL (Supplementary Fig. S1). Equivalent gene expression analysis after co-stimulation with zVAD could not be performed due to the high cytotoxicity after 48 h (compare Fig. 4).

**Fig. 6:**
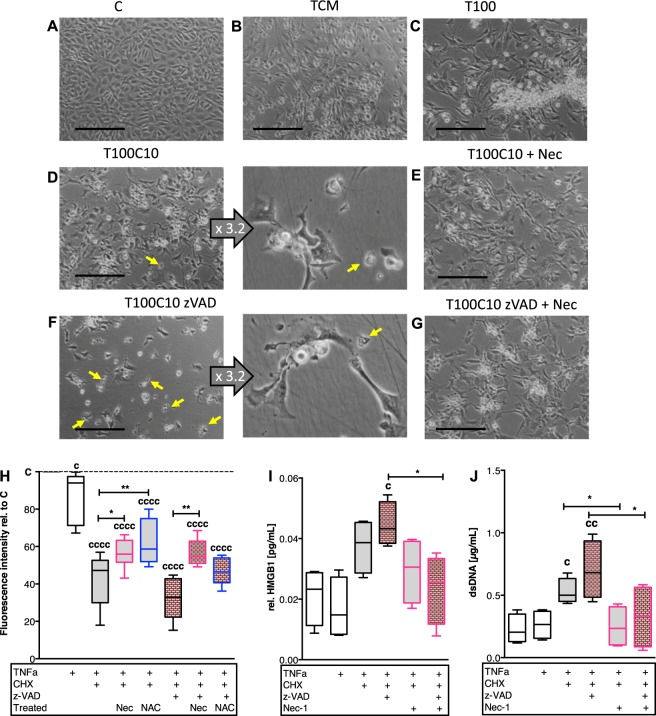
Morphological alteration and DAMP release of chondrocytes undergoing regulated cell death. Isolated chondrocytes were stimulated as follows: **b** trauma-conditioned medium (TCM), **c** 100 ng/mL TNF, **d** 100 ng/mL TNF + 10 µg/mL CHX, and **e** treated with Nec-1, or **f** 100 ng/mL TNF + 10 µg/mL CHX + 20 µM zVAD, and **g** treated with Nec-1 for 24 h. **a** Untreated cells served as control. Yellow arrows indicate cell debris/dead cells. Images were acquired using a 10× and 32× objective (magnification of **d** and **f**; illustrated in the middle columns); the bars represent 200 µm. **h** Cell viability of chondrocytes was evaluated by alamarBlue assay. Fluorescence intensity of treated cells was normalized to the untreated control. **i** Total release of dsDNA into the culture medium was quantified by Hoechst 33258. **j** Amounts of HMGB1 in the culture medium was determined by means of a specific ELISA and normalized to the results of the alamarBlue assay. Statistical analysis was performed by one-way ANOVA, including a Bonferroni posttest (*n* = 4); values are given as boxplots with median and whiskers (min to max). Significant differences between groups were depicted as: [vs C] ^c^*P* < 0.05, ^cc^*P* < 0.01, ^cccc^*P* < 0.0001; [between delineated groups] *P < 0.05, **P < 0.01

Cell morphology was considerably changed after stimulation with TNF with and without CHX (Fig. 6a–i). TNF alone caused a largely roundish cell shape, while addition of CHX led to augmented busting of the cell membrane. Treatment with zVAD rather enhanced the effects of TNF/CHX. Nec-1 alleviated cytotoxicity and sustained the chondrocytes phenotype in all approaches.

Confirming results could be found regarding the cell viability as well as release of dsDNA and HMGB1, respectively (Fig. 6a–c). However, stimulation with TCM did not result in significant necroptosis-associated response (Supplementary Fig. S3).

## Discussion

In terms of regulated cell death, necroptosis is considered as the “dirty” counterpart to apoptosis^8^. The release of intracellular components due to plasma membrane rupture is thought to provoke numerous pathomechanisms, such as inflammation and oxidative stress, which in turn might drive further necroptotic cell death, thus creating a vicious circle. To our knowledge, this is the first study investigating trauma-induced necroptosis in human cartilage and providing evidence for necroptosis-associated markers in highly degenerated OA tissue.

After ex vivo cartilage trauma we found significant cell-protective effects of Nec-1, which were stronger than that of caspase-inhibitor zVAD; besides, the combination of both therapeutics revealed additional effects. Furthermore, Nec-1 administration attenuated trauma-induced gene expression of MLKL as well as PGE2 production in impacted cartilage explants. Altogether, these findings indicate a possible link between trauma and necroptosis. Although it should be noted that Nec-1 has not only been described as potent RIPK1 inhibitor, preventing from necroptotic cell death, but might also reduce autophagy and apoptosis after traumatic injuries^15^, exemplary experiments with the selective MLKL inhibitor NSA confirmed, that a certain number of cells most likely undergo necroptosis after mechanical trauma^20^. In general, several studies verified that NSA and Nec-1 possess equivalent anti-necroptotic potency, despite its different modes of action^21–23^. In the end, a clear delimitation between the different modalities of RIPK1-mediated cell death indeed appears vague^24,25^. Even though zVAD attenuated trauma-induced cell death to some extent, the caspase inhibitor simultaneously enhanced the incidence of double positive cells during live/dead staining and p-MLKL positive cells in IHC. Moreover, zVAD increased the release of NO and PGE2 as well as gene expression of RIPK3 after trauma. These results might represent a shift of trauma-induced, predominant apoptotic cell death towards necroptosis as suspected.

Double stained cells, which were positive for EthD-1 as well as calcein-staining might probably reflect an early necroptotic stage, because the cell membrane of necroptotic cells is thought to be permeabilized by nanopores^12^, which might allow influx of EthD-1, while a certain amount of intracellular esterases still needs to be available to generate the green fluorescent derivate from membrane permeable calcein-AM. In contrast, the cell membrane remains intact during apoptosis, thus preventing permeation of EthD-1. For more comprehensive investigation of necroptotic processes within our models, we included several test series with TNF/CHX stimulation and caspase-inhibitor zVAD. CHX-mediated sensitization of chondrocytes towards TNF-induced cell death has previously been described^16,18^. The co-stimulation allows in vitro investigation of necroptosis as well as RIPK1-dependent apoptosis, though addition of zVAD ensures the inhibition of CASP8 activity and formation of the necrosome, respectively^7,26,27^.

Recently, we reported about the cell-protective effects of NAC after cartilage trauma, which are attributed to its antioxidative and thus anti-apoptotic properties^1^. In the present study we observed that NAC treatment significantly reduced necroptosis-associated gene expression and enhanced the cell viability after trauma and TNF/CHX-induced necroptosis. These findings indicate a strong connection between oxidative stress and initiation of necroptosis. In fact, autophosphorylation of RIPK1 has been shown to be triggered by ROS, due to the interaction with redox-sensitive cysteine residues^28^. In accordance with this, antioxidative treatment with the ROS scavenger NAC has been described to prevent oxidative stress-induced necroptosis of human mesenchymal stem cells exposed to H2O2 and murine hepatocytes after reoxygenation, respectively^29,30^. However, both pro-inflammatory factors and ROS are thought to be required for activation of the necroptotic pathway^28,31^, which we also observed in presence of zVAD, and thus CASP8 inhibition. In these approaches, Nec-1 revealed superior cell-protective effects as compared with NAC even after deprivation of the TNF/CHX stimulation. We concluded that zVAD-mediated execution of necroptotic cell death could rather be prevented by direct inhibition of RIPK1—in other words, Nec-1—than by attenuation of the redox-mediated processes.

Interestingly, the IHC staining revealed that necroptosis marker p-MLKL was predominantly located to the lower part of traumatized and highly degenerated cartilage, while chemically induced MLKL phosphorylation rather occurred at the superficial zone. Moreover, Nec-1 treatment of TNF/CHX/zVAD-stimulated cartilage explants seemed to reverse the anti-apoptotic effects of zVAD, as indicated by increased cleavage of CASP8. Considering the previously described anti-apoptotic effects of Nec-1 mentioned above, this finding appears contradictory. However, Nec-1 has currently been found to increase apoptosis in hepatocytes after sepsis, most likely by enhancing the cleavage of caspase 3 as well as caspase 8 and 9 activities^32^. As the pathways of regulated cell death are very complex and closely interwoven, further research will be needed to understand the underlying mechanisms more comprehensively.

Stimulation of isolated chondrocytes with TCM led to slightly enhanced mRNA levels of MLKL and decreased cell viability. Consequently, the stimulus of trauma-released DAMPs alone was not sufficient to significantly induce necroptotic cell death. However, in line with our ex vivo cartilage data we observed severe morphological changes due to TNF/CHX stimulation, which was not attenuated, but rather worsen by co-stimulation with zVAD. In higher magnification, vacuole-like structures as well as considerable number of dark spots—probably stress granules—were observed within the cytoplasm of the cells. Moreover, many cells were burst as indicated by floating cell membrane residues. Comparable changes of the cellular morphology and loss of membrane integrity were reported by Chen et al. in compression-induced necroptosis of rat nucleus pulposus cells^21^. In our study, loss of membrane integrity was found to be consistent with release of intracellular components as shown for dsDNA and the alarmin HMGB1, which is thought to be a crucial inflammatory mediator in OA disease^33^.

In fact, we found a significant increase of NO release due to TNF/CHX/zVAD stimulation in the cartilage trauma model, which could not be confirmed for PGE2. McKinley et al. reported that CHX reduced the availability of endogenous arachidonic acid, resulting in significant impairment of PGE2 production^34^. However, as discussed above zVAD treatment of impacted cartilage explants considerably increased PGE2 and NO release in absence of CHX, which confirms a possible relation between necroptotic cell death and subsequent inflammation.

Although, Zhang et al. described some evidence for necroptotic cell death after repetitive mechanical overload of mandibular cartilage, the chondrocytes not only expressed RIPK1/3 but also CASP8, complicating the distinction between apoptosis and necroptosis^16^. To circumvent this issue, we included the p-MLKL staining, which is considered as decisive marker of necroptotic events. Even though, the staining of p-MLKL was rather low, we could confirm that trauma as well as CHX/TNF w/ and w/o zVAD co-stimulation increased the number of p-MLKL positive cells, which seemed to be attenuated by Nec-1 and NAC treatment.

As necroptotic cell death exhibited minor importance after single ex vivo trauma, even with addition of TNF-a, we assumed that the mechanical stimulus alone was not sufficient to sensitize the cells towards TNF-induced cell death. Therefore, it is conceivable that the induction of necrosis might require further mediators, missing in our narrowly defined model, i.e., further synovium-derived cytokines or other components present in synovial fluid after joint injury. The fact that necroptosis markers were more pronounced in highly degenerated cartilage tissue as compared with impacted macroscopically intact cartilage might also indicate that necroptosis generally increases in the course of OA development and is a feature of late stage OA. Indeed, besides the early trauma situation, necroptosis might be related to age- and disease-related alteration of cellular control mechanisms, such as the anti-apoptosis regulator cellular FLICE-like inhibitory protein, which facilitates a clear decision between life or death and is highly sensitive towards CHX treatment^35^. In addition, we observed a certain portion of cleaved CASP8-positive cells in macroscopically intact cartilage (control) which can be regarded as a possible indicator of commencing apoptotic cell death in early stage OA. Nevertheless, in consideration of our data we suppose that necroptotic cell death, sooner or later, plays a certain role during OA development, enhancing the release of pro-inflammatory mediators and thus accelerating the degenerative processes. Moreover, CASP8 inhibition and subsequent shift of the cell death modality might have rather detrimental effects, which has not been considered so far.

The main limitation of this study is the exclusion of certain physiological parameter such as synovial components and repetitive loading which might influence the modality of cell death after cartilage trauma. We discussed that additional stimuli might be involved in trauma-induced necroptosis so that the identification of those factors should be addressed in future studies.

Taken together, our study provides novel evidence for the involvement of necroptosis in OA disease and elucidates a possible link between cartilage injury and necroptotic processes, though, the mechanical stimulus alone might not be a sole trigger for necroptosis. It is highly probable, that necroptotic cell death depends on additional extrinsic and intracellular mediators, besides oxidative stress and cytokines, modulating the cell fate. Elucidating the responsible factors and molecular mechanisms involved may lead to novel therapeutic strategies after cartilage trauma.

## Supplementary information

Titration of the appropriate Nec-1 concentration in TNF/CHX-stimulated chondrocytes

Gene expression analysis of cartilage explants

Stimulation of isolated chondrocytes with cartilage-/ trauma-conditioned medium
