# Supplementary material for: Evidence of necroptosis in osteoarthritic disease: investigation of blunt mechanical impact as possible trigger in regulated necrosis
Source: Cell Death Dis. 2019 Sep 17;10(10):683. doi: 10.1038/s41419-019-1930-5 (PMC6746800; doi:10.1038/s41419-019-1930-5)
Supplement: Supplementary file 1 — Titration of the appropriate Nec-1 concentration in TNF/CHX-stimulated chondrocytes [file 41419_2019_1930_MOESM1_ESM.docx]

**Supplementary Figure 1**

**A**

**D**

**B**

**E**

**C**

**Figure S1: Titration of the appropriate Nec-1 concentration in TNF/CHX-stimulated chondrocytes.** Appropriate concentration of Nec-1 (pink border color) was determined in isolated chondrocytes stimulated with TNF-a (100 ng/mL) and CHX (10 µg/mL) by means of (**A**) alamarBlue cell proliferation and cytotoxicity assay as well as (**B-E**) gene expression analysis. DMSO control (gray border color) was included to evaluate possible influence of the solvent used for Nec-1 and CHX.
