# Supplementary material for: Evidence of necroptosis in osteoarthritic disease: investigation of blunt mechanical impact as possible trigger in regulated necrosis
Source: Cell Death Dis. 2019 Sep 17;10(10):683. doi: 10.1038/s41419-019-1930-5 (PMC6746800; doi:10.1038/s41419-019-1930-5)
Supplement: Supplementary file 2 — Gene expression analysis of cartilage explants [file 41419_2019_1930_MOESM2_ESM.docx]

**Supplementary Figure 2**

**4d (continuously) stimulated**

**24h stimulated**

**4d (continuously) stimulated**

**24h stimulated**

**4d (continuously) stimulated**

**24h stimulated**

**C**

**B**

**A**

**Figure S2: Gene expression analysis of cartilage explants.** Chemical induction of necroptosis by TNF/CHX stimulation was performed in different concentrations as well as exposure times (24h= deprived; 4d=continuously). Gene expression of (**A**) RIPK1, (**B**) RIPK3 and (**C**) MLKL was performed 4d after impact. Blank symbols= unimpacted, black symbols= impacted, pink border color= Nec-1 treated (continuously), blue border color= NAC treated (continuously); n ≥ 3.
