# Supplementary material for: Evidence of necroptosis in osteoarthritic disease: investigation of blunt mechanical impact as possible trigger in regulated necrosis
Source: Cell Death Dis. 2019 Sep 17;10(10):683. doi: 10.1038/s41419-019-1930-5 (PMC6746800; doi:10.1038/s41419-019-1930-5)
Supplement: Supplementary file 3 — Stimulation of isolated chondrocytes with cartilage-/ trauma-conditioned medium [file 41419_2019_1930_MOESM3_ESM.docx]

**Supplementary Figure 3**

**B**

**A**

**Figure S3: Stimulation of isolated chondrocytes with cartilage-/ trauma-conditioned medium.** Isolated chondrocytes were stimulated with cartilage-/ trauma conditioned medium (1:1 conditioned (serum-free) medium/ serum-containing medium; 5% FCS (v/v)). 48h after stimulation, (**A**) cell viability and (**B**) gene expression of necroptosis- and apoptosis-associated markers were determined. Cell viability was measured by alamarBlue cell proliferation and cytotoxicity assay; n ≥ 4.
